# Clinical and Biochemical Factors Associated With Hospitalization and Intensive Care Unit Admission Among Pregnant Women With Severe Psoriasis: A Retrospective Cohort Study

**DOI:** 10.7759/cureus.114717

**Published:** 2026-08-18

**Authors:** Hafsa Abid, Shahan Temoor Farooq, Sammiya Uraneb, Hina Athif, Muhammad Athif Akram, Naveed Shuja

**Affiliations:** 1 Department of Clinical Medicine, Frimley Health NHS Foundation Trust, Slough, GBR; 2 Department of Biochemistry, Institute of Molecular Biology and Biotechnology (IMBB), The University of Lahore, Lahore, PAK; 3 Department of Anesthesiology and Intensive Care, Allama Iqbal Medical College, Lahore, PAK; 4 Department of Anesthesiology and Intensive Care, Abu Umara Medical and Dental College, Lahore, PAK; 5 Department of Anesthesiology and Intensive Care, Ali Fatima Teaching Hospital, Lahore, PAK; 6 Department of Biochemistry, Continental Medical College (CMC), Lahore, PAK

**Keywords:** c-reactive protein, hospitalization, hypoalbuminemia, intensive care unit, pregnancy, severe psoriasis

## Abstract

Background and objective

Severe psoriasis during pregnancy may be complicated by systemic inflammation, biochemical abnormalities, infection, and organ dysfunction requiring hospitalization or intensive care. Factors associated with escalation of care remain insufficiently characterized. The main objective of this study is to identify clinical and biochemical factors associated with hospitalization among pregnant women with severe psoriasis and explore factors associated with intensive care unit (ICU) admission.

Methods

This retrospective cohort study included 100 pregnant women with dermatologist-confirmed severe psoriasis treated at Continental Medical College, Lahore, Pakistan, from February to June 2026. Severe psoriasis was defined by Psoriasis Area and Severity Index ≥10, body surface area involvement ≥10%, generalized pustular psoriasis, or erythrodermic psoriasis. Clinical and biochemical findings recorded at presentation were compared between hospitalized and non-hospitalized participants. Multivariable logistic regression assessed factors independently associated with hospitalization. ICU analyses were exploratory because only 17 participants required intensive care.

Results

Fifty-eight participants were hospitalized, including 17 admitted to the ICU, while 42 were managed without admission. Generalized pustular or erythrodermic psoriasis (adjusted odds ratio (aOR), 4.16; 95% confidence interval (CI), 1.49-11.63), hypoalbuminemia (aOR, 3.42; 95% CI, 1.38-8.47), and elevated C-reactive protein (CRP) (aOR, 2.89; 95% CI, 1.21-6.91) were independently associated with hospitalization. Exploratory ICU analyses showed associations with systemic infection, acute kidney injury, hepatic dysfunction, and marked leukocytosis.

Conclusion

Severe systemic psoriasis phenotypes, elevated CRP, and hypoalbuminemia were independently associated with hospitalization. ICU findings were exploratory. These factors may support closer monitoring and multidisciplinary assessment but require external validation before use for clinical prediction or formal risk stratification.

## Introduction

Psoriasis is a chronic immune-mediated inflammatory disease characterized by recurrent cutaneous inflammation and, in some patients, systemic manifestations [1]. Its course during pregnancy is variable. Although pregnancy-related immunological changes may improve disease activity in some women, others may experience persistent, worsening, or newly diagnosed severe psoriasis [2]. Severe plaque psoriasis, generalized pustular psoriasis, and erythrodermic psoriasis may be accompanied by fever, disruption of the epidermal barrier, dehydration, electrolyte disturbances, secondary infection, nutritional depletion, and multiorgan dysfunction [3]. Such complications may substantially increase maternal morbidity and necessitate hospitalization or, in severe cases, admission to an intensive care unit (ICU) [4].

Clinical assessment of severe psoriasis during pregnancy is complicated by the physiological and biochemical changes associated with gestation [5]. Hemoglobin and serum albumin concentrations may decline, while leukocyte counts can increase even in the absence of infection. Pregnancy also alters renal filtration, hepatic metabolism, fluid balance, and inflammatory responses [2]. Consequently, inflammatory markers, liver enzymes, renal function tests, serum proteins, and electrolyte concentrations should be interpreted within the physiological context of pregnancy. Nevertheless, marked or progressively worsening abnormalities may accompany severe systemic inflammation, infection, reduced physiological reserve, or organ dysfunction [6].

Hospitalization may be required for pregnant women with severe psoriasis who develop rapidly progressive skin involvement, generalized pustulation, erythroderma, uncontrolled symptoms, dehydration, fever, suspected infection, treatment-related complications, or a need for close maternal and fetal surveillance [7,8]. More severe clinical deterioration may be accompanied by major electrolyte disturbances, respiratory compromise, extensive fluid or protein loss, acute kidney injury (AKI), hepatic dysfunction, sepsis, or hemodynamic instability requiring intensive monitoring and supportive care. Recognition of clinical and biochemical abnormalities associated with greater care requirements may facilitate timely multidisciplinary assessment involving dermatology, obstetrics, internal medicine, infectious disease, and critical care specialists [9].

Previous studies of psoriasis in pregnancy have primarily examined changes in disease activity, medication safety, fertility, obstetric complications, and neonatal outcomes [10]. Comparatively limited evidence is available regarding the clinical and biochemical characteristics associated with hospitalization or ICU admission among pregnant women with severe psoriasis. Routinely available laboratory parameters, including C-reactive protein (CRP), leukocyte count, serum albumin, liver enzymes, serum creatinine, and electrolytes, have also been insufficiently evaluated in relation to these care outcomes in this population [11].

Accordingly, this study primarily evaluated the clinical and biochemical factors associated with hospitalization among pregnant women with severe psoriasis. A secondary exploratory analysis examined factors associated with ICU admission. We hypothesized that generalized pustular or erythrodermic psoriasis, systemic inflammation, hypoalbuminemia, infection, and biochemical evidence of hepatic or renal dysfunction would be associated with hospitalization, while ICU-related associations were considered exploratory because of the limited number of intensive care events [12].

## Materials and methods

Study design and setting

This retrospective cohort study was conducted at Continental Medical College, Lahore, Pakistan, and included pregnant women presenting with severe psoriasis between February 2026 and June 2026. The institution has a high clinical patient flow, and relevant clinical and biochemical data were readily available across dermatology, obstetric, emergency department, inpatient, laboratory, and critical care records, facilitating consecutive identification of eligible cases during the study period. The primary analysis evaluated clinical and biochemical factors associated with hospitalization, while ICU admission was examined as a secondary exploratory outcome because of the limited number of intensive care events.

Study population

Eligible participants were pregnant women aged 18 years or older with dermatologist-confirmed severe psoriasis. Severe psoriasis was defined objectively by the presence of at least one of the following: a Psoriasis Area and Severity Index (PASI) score ≥10, body surface area (BSA) involvement ≥10%, generalized pustular psoriasis, or erythrodermic psoriasis. The requirement for urgent systemic assessment or treatment was not used as an independent severity criterion to avoid incorporating a treatment-related decision into the eligibility definition.

Women were excluded if they had mild or localized psoriasis, an uncertain diagnosis, incomplete clinical or laboratory information required for the analysis, or an unavailable hospitalization outcome. Additional exclusion criteria were end-stage kidney disease, decompensated chronic liver disease, active malignancy, or another systemic inflammatory disorder likely to substantially influence the biochemical parameters under investigation. For women with multiple eligible presentations during the study period, only the first presentation was included.

Sampling and data collection

Consecutive sampling was applied. A total of 112 potentially eligible medical records were screened. Of the 112 patients with psoriasis, 12 were excluded due to incomplete clinical or biochemical data, unclear psoriasis diagnosis, or underlying compromised organ status, leading to a final cohort of 100 patients. Information was collected using a pre-designed data collection form from dermatology, obstetric, emergency department, inpatient, laboratory, and critical care records. Variables recorded included age, parity, gestational age, medical and obstetric comorbidities, previous hospitalization, previous systemic treatment, treatment interruption during pregnancy, fever, and documented evidence of infection. Psoriasis treatment information available before presentation was also reviewed, as exposure to or stopping of treatment may affect clinical presentation and biochemical measurements.

Laboratory parameters were hemoglobin, total leukocytes, CRP, serum albumin, alanine aminotransferase (ALT), aspartate aminotransferase (AST), serum creatinine, sodium, potassium, and corrected calcium. Pre-index measurements were used when taken at the index presentation and before significant inpatient treatment. These variables were not considered to be validated predictors, but rather factors that were thought to be associated with subsequent care requirements, since the retrospective records did not allow the exact interval between each laboratory measurement and the clinical decision to hospitalize to be uniformly reconstructed. ICU analyses were thus also deemed exploratory, as opposed to predictive. Records that did not contain the necessary information to determine eligibility or primary hospitalization outcome were excluded at screening. No statistical imputation was performed; analyses were conducted using complete available cases for each variable.

Operational definitions

Hypoalbuminemia was defined as serum albumin <3.5 g/dL. Elevated CRP was defined as CRP >10 mg/L, and this threshold was applied consistently throughout categorical analyses. There was no separate outcome-derived CRP threshold. A raised total leucocyte count of >20.0 × 10⁹/L was defined as marked leukocytosis. This increased cut-off was chosen to minimize potential misclassification due to leukocytosis during pregnancy. Hepatic dysfunction was defined as an ALT or AST concentration greater than twice the pregnancy-appropriate upper reference limit. AKI was defined based on Kidney Disease: Improving Global Outcomes (KDIGO) serum creatinine criteria. Electrolyte disturbance was considered to be a serum sodium or potassium level or corrected calcium level outside of the normal laboratory range. Subsequent treatment of an electrolyte abnormality was not used as part of its definition.

Systemic infection was determined irrespective of antimicrobial therapy and included a clinical infectious syndrome or an infectious focus, as appropriate, with positive microbiological culture or compatible radiological findings. Prescription of intravenous antimicrobial therapy alone was not considered sufficient to classify systemic infection. For regression analysis, generalized pustular psoriasis and erythrodermic psoriasis were classified together as severe systemic psoriasis phenotypes, given that they are both inflammatory types of psoriasis that are extensive.

Outcome measures

The primary outcome was hospitalization during the index presentation. Hospitalization was defined solely as admission to hospital for at least 24 hours. Clinical reasons for admission, including systemic treatment, maternal monitoring, fetal surveillance, suspected infection, biochemical abnormalities, or organ dysfunction, were recorded separately and were not incorporated into the outcome definition. Because this was a retrospective observational study, the research protocol did not determine whether a patient was hospitalized. Admission represented the care-level decision documented during routine clinical practice by the responsible treating team.

The secondary outcome was actual admission or transfer to an ICU or high-dependency unit during the index hospitalization. ICU admission was defined according to the level of care received rather than by the presence of infection, AKI, hepatic dysfunction, electrolyte abnormalities, or other factors subsequently examined in relation to ICU care. Reasons for ICU transfer were recorded separately. Each participant was classified according to the highest level of care received during the index presentation.

Statistical analysis

Continuous variables were summarized as mean ± standard deviation when approximately normally distributed and as median with interquartile range when non-normally distributed. Categorical variables were presented as frequencies and percentages. Hospitalized and non-hospitalized participants were compared using the independent-samples t-test or Mann-Whitney U test for continuous variables, as appropriate, and the chi-square test or Fisher's exact test for categorical variables. Univariable binary logistic regression was used to estimate associations between candidate factors and hospitalization. Covariates for the multivariable hospitalization analysis were selected according to clinical and biological relevance and their availability at the index presentation rather than solely based on univariable statistical significance. Collinearity among candidate variables was assessed before model construction, and variables representing substantially overlapping clinical constructs were not entered simultaneously.

Clinically prespecified thresholds were used for elevated CRP, hypoalbuminemia, and marked leukocytosis. Corresponding continuous laboratory values were also reported in descriptive analyses to preserve information regarding their distributions and to avoid interpretation based exclusively on dichotomized measurements. Multivariable binary logistic regression was used to estimate independent associations with hospitalization. Results were reported as unadjusted and adjusted odds ratios (ORs) with 95% confidence intervals (CIs). Model discrimination was described using the area under the receiver operating characteristic curve, while the Hosmer-Lemeshow goodness-of-fit test was used to assess calibration. These measures were considered descriptive characteristics of the fitted association model and were not interpreted as external validation or evidence of a clinically validated prediction tool.

Because only 17 participants required ICU care, ICU analyses were explicitly considered exploratory and hypothesis-generating. To reduce overparameterization and model instability, no multivariable ICU prediction model was used in the revised analysis. Ward-based and ICU-treated participants were compared using Fisher's exact test for categorical variables and the Mann-Whitney U test for continuous variables, as appropriate. Univariable association estimates could be reported for selected clinically relevant factors, but no conclusions regarding independent prediction of ICU admission were made. All statistical tests were two-sided, and p<0.05 was considered statistically significant. Statistical analyses were performed using IBM SPSS Statistics for Windows, Version 27.0 (Released 2019; IBM Corp., Armonk, NY, USA).

Ethical considerations

The study was conducted in accordance with the principles of the Declaration of Helsinki and was approved by the Institutional Review Committee of Continental Medical College, Lahore, Pakistan (reference number: 87-IRB-CMC). The requirement for informed consent was waived because the study involved retrospective review of de-identified medical records. All direct patient identifiers were removed before data analysis.

## Results

Participant characteristics

A total of 112 medical records were screened for eligibility. Twelve records were excluded because of incomplete clinical or biochemical information, uncertain classification of psoriasis, pre-existing advanced organ disease, or unavailable hospitalization outcomes. The final cohort therefore comprised 100 pregnant women with severe psoriasis.

The mean age of the participants was 29.8 ± 5.2 years, and the mean gestational age at presentation was 25.6 ± 8.1 weeks. Severe plaque psoriasis was present in 56 (56.0%) participants, generalized pustular psoriasis in 27 (27.0%), and erythrodermic psoriasis in 17 (17.0%). Overall, 58 (58.0%) women were hospitalized, whereas 42 (42.0%) were managed without hospitalization. Among hospitalized participants, 17 required ICU admission, representing 17.0% of the entire cohort and 29.3% of hospitalized women.

Age, gestational age, third-trimester presentation, and primigravida status did not differ significantly according to hospitalization status. Generalized pustular or erythrodermic psoriasis was substantially more frequent among hospitalized women than among those managed without hospitalization (58.6% vs. 23.8%; p<0.001). Fever at presentation (44.8% vs. 11.9%; p<0.001), treatment interruption during pregnancy (48.3% vs. 26.2%; p=0.026), and systemic infection (24.1% vs. 4.8%; p=0.009) were also more common among hospitalized participants.

Significant biochemical differences were observed between the two groups. Hospitalized participants had lower hemoglobin concentrations (10.1 ± 1.4 vs. 11.2 ± 1.2 g/dL; p<0.001) and serum albumin concentrations (3.0 ± 0.5 vs. 3.7 ± 0.5 g/dL; p<0.001). They also had higher median leukocyte counts (16.1 vs. 10.9 × 10⁹/L; p<0.001), CRP concentrations (38 vs. 12 mg/L; p<0.001), ALT concentrations (36 vs. 21 U/L; p=0.002), and serum creatinine levels (0.81 vs. 0.64 mg/dL; p<0.001).

Hypoalbuminemia occurred in 65.5% of hospitalized participants compared with 23.8% of non-hospitalized participants (p<0.001). Marked leukocytosis, hepatic dysfunction, AKI, and electrolyte disturbance were likewise significantly more frequent among hospitalized women. The complete comparison is presented in Table 1.

**Table 1:** Clinical and biochemical characteristics according to hospitalization status Independent-samples t-test, Mann-Whitney U test, chi-square test, or Fisher's exact test was used as appropriate. A two-sided p-value <0.05 was considered statistically significant. SD: standard deviation; IQR: interquartile range; CRP: C-reactive protein

Characteristic	Total (n=100)	Not hospitalized (n=42)	Hospitalized (n=58)	p-value
Age, years, mean ± SD	29.8 ± 5.2	29.1 ± 5.0	30.3 ± 5.3	0.25
Gestational age, weeks, mean ± SD	25.6 ± 8.1	24.8 ± 8.0	26.2 ± 8.2	0.39
Third-trimester presentation, n (%)	42 (42.0)	15 (35.7)	27 (46.6)	0.28
Primigravida, n (%)	37 (37.0)	17 (40.5)	20 (34.5)	0.54
Severe plaque psoriasis, n (%)	56 (56.0)	32 (76.2)	24 (41.4)	<0.001
Generalized pustular psoriasis, n (%)	27 (27.0)	7 (16.7)	20 (34.5)	0.047
Erythrodermic psoriasis, n (%)	17 (17.0)	3 (7.1)	14 (24.1)	0.026
Pustular or erythrodermic phenotype, n (%)	44 (44.0)	10 (23.8)	34 (58.6)	<0.001
Fever at presentation, n (%)	31 (31.0)	5 (11.9)	26 (44.8)	<0.001
Treatment interruption, n (%)	39 (39.0)	11 (26.2)	28 (48.3)	0.026
Systemic infection, n (%)	16 (16.0)	2 (4.8)	14 (24.1)	0.009
Hemoglobin, g/dL, mean ± SD	10.6 ± 1.4	11.2 ± 1.2	10.1 ± 1.4	<0.001
Leukocyte count, ×10⁹/L, median (IQR)	13.6 (10.2-18.7)	10.9 (8.8-14.1)	16.1 (11.9-21.4)	<0.001
Marked leukocytosis, n (%)	21 (21.0)	3 (7.1)	18 (31.0)	0.004
CRP, mg/L, median (IQR)	24 (9-58)	12 (5-23)	38 (18-82)	<0.001
Elevated CRP, n (%)	61 (61.0)	15 (35.7)	46 (79.3)	<0.001
Serum albumin, g/dL, mean ± SD	3.3 ± 0.6	3.7 ± 0.5	3.0 ± 0.5	<0.001
Hypoalbuminemia, n (%)	48 (48.0)	10 (23.8)	38 (65.5)	<0.001
Alanine aminotransferase, U/L, median (IQR)	27 (18-49)	21 (16-31)	36 (21-67)	0.002
Hepatic dysfunction, n (%)	23 (23.0)	4 (9.5)	19 (32.8)	0.007
Serum creatinine, mg/dL, median (IQR)	0.72 (0.59-0.91)	0.64 (0.55-0.75)	0.81 (0.64-1.02)	<0.001
Acute kidney injury, n (%)	14 (14.0)	2 (4.8)	12 (20.7)	0.024
Electrolyte disturbance, n (%)	18 (18.0)	3 (7.1)	15 (25.9)	0.017

Factors associated with hospitalization

Univariable logistic regression demonstrated significant associations between hospitalization and treatment interruption, generalized pustular or erythrodermic psoriasis, elevated CRP, hypoalbuminemia, marked leukocytosis, hepatic dysfunction, AKI, and electrolyte disturbance. Age and third-trimester presentation were not significantly associated with hospitalization.

After multivariable adjustment, generalized pustular or erythrodermic psoriasis was independently associated with hospitalization compared with severe plaque psoriasis (aOR, 4.16; 95% CI, 1.49-11.63; p=0.007). Hypoalbuminemia was also independently associated with hospitalization (aOR, 3.42; 95% CI, 1.38-8.47; p=0.008), as was elevated CRP (aOR, 2.89; 95% CI, 1.21-6.91; p=0.017).

Treatment interruption, marked leukocytosis, hepatic dysfunction, AKI, and electrolyte disturbance showed significant univariable associations but did not retain statistically significant independent associations after adjustment. The regression findings are summarized in Table 2.

**Table 2:** Logistic regression analysis of factors associated with hospitalization Univariable and multivariable binary logistic regression analyses were performed. Categorical laboratory thresholds were prespecified in the Methods. A two-sided p-value <0.05 was considered statistically significant. OR: odds ratio; CI: confidence interval; CRP: C-reactive protein

Factor	Unadjusted OR (95% CI)	p-value	Adjusted OR (95% CI)	p-value
Age, per year	1.05 (0.97-1.13)	0.25	1.03 (0.94-1.13)	0.51
Third-trimester presentation	1.57 (0.70-3.52)	0.28	1.28 (0.48-3.41)	0.62
Treatment interruption	2.63 (1.12-6.18)	0.027	1.88 (0.68-5.19)	0.22
Pustular or erythrodermic phenotype vs. severe plaque psoriasis	4.53 (1.88-10.92)	<0.001	4.16 (1.49-11.63)	0.007
Elevated CRP	6.91 (2.82-16.93)	<0.001	2.89 (1.21-6.91)	0.017
Hypoalbuminemia	6.08 (2.46-15.01)	<0.001	3.42 (1.38-8.47)	0.008
Marked leukocytosis	5.85 (1.60-21.40)	0.008	2.18 (0.54-8.74)	0.27
Hepatic dysfunction	4.63 (1.46-14.66)	0.009	2.21 (0.59-8.26)	0.24
Acute kidney injury	5.21 (1.10-24.66)	0.038	2.49 (0.45-13.72)	0.30
Electrolyte disturbance	4.54 (1.23-16.72)	0.023	2.06 (0.46-9.15)	0.34

The fitted hospitalization model demonstrated good discrimination, with an area under the receiver operating characteristic curve of 0.86 (95% CI, 0.78-0.93). The Hosmer-Lemeshow goodness-of-fit test indicated satisfactory calibration (p=0.61). These measures describe model performance within the present cohort and should not be interpreted as external validation or evidence of a clinically validated prediction model.

Exploratory analysis of ICU admission

Among the 58 hospitalized participants, 17 required ICU admission, whereas 41 received ward-based care. Given the small number of ICU events, these comparisons were considered exploratory and hypothesis-generating. No multivariable ICU model was interpreted in the revised analysis.

Generalized pustular or erythrodermic psoriasis was present in 14 of 17 ICU-treated participants (82.4%) compared with 20 of 41 participants receiving ward-based care (48.8%). Marked leukocytosis was present in 76.5% of ICU-treated participants compared with 12.2% of ward-treated participants. Similarly, hepatic dysfunction occurred in 64.7% versus 19.5%, AKI in 52.9% versus 7.3%, electrolyte disturbance in 52.9% versus 14.6%, and systemic infection in 70.6% versus 4.9%, respectively.

Exploratory comparisons demonstrated statistically significant differences between ICU and ward-based participants for pustular or erythrodermic phenotype (p=0.022), marked leukocytosis (p<0.001), hepatic dysfunction (p=0.002), AKI (p<0.001), electrolyte disturbance (p=0.006), and systemic infection (p<0.001). Because these findings were derived from only 17 ICU events, they should be interpreted as associations requiring confirmation in larger cohorts rather than as independent predictive effects.

The previously reported ICU-specific CRP threshold of >50 mg/L was removed because it had not been prespecified in the Methods and therefore could represent outcome-dependent cut-point selection. CRP was reported continuously and using the prespecified >10 mg/L threshold in the primary hospitalization analysis. The median hospital stay was 11 days (IQR, 8-16 days) among ICU-treated participants compared with five days (IQR, 3-7 days) among women receiving ward-based care (p<0.001). The exploratory ICU comparisons are summarized in Table 3.

**Table 3:** Exploratory comparison of ward-based and intensive care unit treatment among hospitalized participants Fisher's exact test was used for categorical comparisons because of the small number of ICU events and sparse cell counts. Hospital stay was compared using the Mann-Whitney U test. ICU findings were considered exploratory and hypothesis-generating. ICU: intensive care unit; IQR: interquartile range

Factor	Ward care (n=41), n (%)	ICU care (n=17), n (%)	p-value
Pustular or erythrodermic phenotype	20 (48.8)	14 (82.4)	0.022
Marked leukocytosis	5 (12.2)	13 (76.5)	<0.001
Hepatic dysfunction	8 (19.5)	11 (64.7)	0.002
Acute kidney injury	3 (7.3)	9 (52.9)	<0.001
Electrolyte disturbance	6 (14.6)	9 (52.9)	0.006
Systemic infection	2 (4.9)	12 (70.6)	<0.001
Hospital stay, days, median (IQR)	5 (3-7)	11 (8-16)	<0.001

No maternal deaths occurred during the index hospitalization, despite the substantial burden of severe disease and the need for intensive care among a subset of participants.

## Discussion

This retrospective cohort study identified several clinical and biochemical factors associated with hospitalization among pregnant women with severe psoriasis. More than half of the participants were hospitalized, and 17.0% of the overall cohort required ICU admission. After multivariable adjustment, generalized pustular or erythrodermic psoriasis, elevated CRP, and hypoalbuminemia remained independently associated with hospitalization. Among hospitalized women, systemic infection, marked leukocytosis, hepatic dysfunction, AKI, electrolyte disturbance, and generalized pustular or erythrodermic phenotype were more frequent among those requiring ICU care. Severe psoriasis during pregnancy may involve systemic inflammation and clinically important maternal complications requiring coordinated dermatological and obstetric management [1,2]. Given the limited number of ICU events in the present cohort, these findings should be interpreted as exploratory and hypothesis-generating rather than as evidence of independent prediction [3].

The association between generalized pustular or erythrodermic psoriasis and hospitalization is clinically plausible [4]. These phenotypes represent extensive systemic inflammatory forms of psoriasis and may be accompanied by disruption of the epidermal barrier, fever, fluid loss, electrolyte abnormalities, protein depletion, and secondary infection [3]. Pregnancy may further modify physiological reserve through plasma-volume expansion, altered immune responses, and increased metabolic requirements [5]. Consequently, women with severe systemic psoriasis phenotypes may require closer maternal assessment, systemic treatment, biochemical monitoring, and coordinated obstetric surveillance [5,6].

Elevated CRP was independently associated with hospitalization in the present cohort. CRP is a widely available but nonspecific marker of systemic inflammation and may increase in response to extensive cutaneous inflammation, infection, or a combination of both [7]. Inflammatory biomarkers in psoriasis should therefore be interpreted in conjunction with the overall clinical picture rather than as isolated measures of disease severity [7,8]. CRP may provide the greatest clinical information when considered together with psoriasis phenotype, fever, evidence of infection, leukocyte count, serum albumin, renal function, and hepatic parameters [9]. The present findings demonstrate an association with hospitalization but do not establish a CRP threshold capable of independently predicting admission [4].

Hypoalbuminemia was also independently associated with hospitalization. Serum albumin concentrations normally decline during pregnancy as a consequence of plasma-volume expansion, whereas more pronounced reductions may occur with systemic inflammation, inadequate nutritional intake, decreased hepatic synthesis, renal loss, or protein loss through extensively inflamed skin [10]. Inflammatory states can additionally alter hepatic albumin synthesis and increase vascular permeability, thereby contributing to lower circulating albumin concentrations [11]. Hypoalbuminemia may therefore reflect both inflammatory burden and reduced physiological reserve, although serum albumin should not be interpreted as an isolated marker of psoriasis severity [11,12].

In the exploratory ICU analysis, marked leukocytosis was substantially more frequent among ICU-treated participants than among women receiving ward-based care. Leukocyte counts increase physiologically during pregnancy, particularly during later gestation, and modest elevations therefore do not necessarily indicate pathological inflammation or infection [13]. Pregnancy-specific hematological changes must be considered when interpreting leukocyte concentrations in acutely unwell patients [14]. A comparatively high threshold for marked leukocytosis was therefore used in this study to reduce possible misclassification related to physiological leukocytosis. Even so, leukocyte count should be interpreted together with clinical findings, microbiological investigations, inflammatory markers, and evidence of organ dysfunction [9].

Systemic infection was also substantially more frequent among participants requiring intensive care. Severe inflammatory skin disease may increase susceptibility to infection because of disruption of the cutaneous barrier, excoriation, extensive skin involvement, chronic inflammation, and exposure to immunomodulatory therapies [15]. Distinguishing infection from sterile inflammation may be particularly challenging in generalized pustular psoriasis because fever, leukocytosis, and elevated inflammatory markers can occur in both conditions [15,16]. Microbiological investigation, assessment for a focal source of infection, repeated clinical evaluation, and appropriate antimicrobial therapy remain important when infection is clinically suspected. In the present study, systemic infection was defined independently of antimicrobial prescription to minimize treatment-related classification bias [11].

AKI and hepatic dysfunction were also more frequent among participants requiring ICU care. Renal dysfunction in severely unwell pregnant patients may occur in association with dehydration, systemic infection, hemodynamic instability, electrolyte abnormalities, or medication-related toxicity [17]. Interpretation of serum creatinine during pregnancy requires particular caution because physiological increases in glomerular filtration generally result in lower baseline creatinine concentrations than in nonpregnant adults [18]. Consequently, relatively modest increases may represent clinically important renal impairment. Similarly, elevated hepatic enzymes may accompany systemic inflammation, infection, drug-related toxicity, or pregnancy-specific hepatic disorders and should prompt appropriate clinical evaluation [19].

The present findings support a comprehensive approach to the assessment of pregnant women with severe psoriasis. Evaluation should extend beyond the extent of cutaneous involvement and include assessment for systemic inflammation, infection, protein depletion, electrolyte disturbances, and evidence of hepatic or renal dysfunction [17,19]. Routinely available investigations such as a complete blood count, CRP, serum albumin, liver enzymes, renal function tests, and serum electrolytes may provide useful complementary clinical information [19]. Early multidisciplinary involvement of dermatology, obstetrics, internal medicine, infectious disease, and critical care services may be appropriate when severe systemic manifestations or organ dysfunction are present [17,20]. However, these findings should not be interpreted as constituting a validated triage or risk-stratification algorithm [5].

Treatment before presentation may influence both disease activity and biochemical measurements. Treatment interruption during pregnancy was more frequent among hospitalized participants in the present cohort, although it did not retain an independent association after multivariable adjustment. Topical corticosteroids, conventional systemic therapies, biologic agents, and withdrawal or interruption of treatment may influence inflammatory activity, susceptibility to infection, liver enzymes, blood counts, and other biochemical parameters [10,15]. Medication selection during pregnancy additionally requires consideration of maternal disease control and potential fetal effects [15,17]. Detailed information regarding specific medication type, dose, duration, and timing was not consistently available in the retrospective records; therefore, residual confounding related to treatment exposure cannot be excluded [11].

These findings may be particularly relevant in healthcare settings where access to coordinated dermatological, obstetric, laboratory, microbiological, and critical care services is limited. In some dermatologic emergencies of pregnancy, prompt multidisciplinary cooperation has to be established, and delays in the diagnosis of infection, dehydration, electrolyte disturbances, or progressive organ dysfunction may add to the difficulty of management [16,18]. Escalation-of-care decisions and clinical outcomes can therefore be affected by the availability of resources and organization of institutions [19]. Biochemical parameters are routinely available and may be used in conjunction with careful clinical judgment as part of an integrated clinical assessment in resource-limited settings [5].

There are a number of strengths in this study. It has studied an under-researched group of patients at the nexus of dermatology and maternal-fetal medicine, employed consecutive identification of eligible records, and included clinical and biochemical parameters that are easily and routinely obtained in clinical practice. The literature on psoriasis in pregnancy to date has mainly addressed psoriasis disease activity, therapeutic safety, and obstetric outcome, while available data on escalation of maternal care are relatively scarce [1,2,10]. Also, the difference between the primary end point of hospitalization and the exploratory secondary end point of ICU admission permits the primary end point results to be considered in isolation from the smaller ICU subset [12].

The results are subject to several limitations. The retrospective nature relied on the completeness and accuracy of available medical records, and the sample was relatively small and from a single center, potentially impacting the generalizability to other populations with distinct clinical and healthcare systems [11,15]. Furthermore, only 17 participants required ICU admission, making ICU-related comparisons statistically imprecise and vulnerable to instability. Analyses based on a small number of events may yield unstable and imprecise estimates; therefore, the ICU findings should be interpreted as exploratory and hypothesis-generating rather than confirmatory [3,14].

Hospitalization and ICU admission are markers of the quality of the care process and not solely biological measures of how severe a disease is. Clinician judgment, institutional admission criteria, bed availability, demand for fetal monitoring, availability of specialists, availability of treatment resources, and local healthcare practices may all affect the decision for admission [6,19]. These context effects may have an impact on apparent associations without influencing biological severity and may constrain causal interpretation and external generalizability [19]. Therefore, the relationships found in this study cannot be assumed to mean the same escalation thresholds in other institutions [20].

There was a lack of detailed description of specific pre-admission medication exposures. Treatment interruption was mentioned, but details on biologic agents, topical corticosteroids, systemic therapy, cumulative exposure to treatment, and timing of exposure in relation to presentation were not always available [15]. Potential residual confounding is possible because psoriasis treatments can alter inflammatory levels, the risk of infection, and liver biochemical markers and hematological indicators [16]. Additionally, nutritional status, socioeconomic factors, and some obstetric confounders were not fully available and should be considered in future prospective studies [17,18].

Granular analyses according to specific gestational-week ranges were limited by the retrospective nature and size of the dataset. Gestational age may influence maternal physiology, laboratory reference ranges, treatment decisions, fetal surveillance requirements, and thresholds for hospitalization [5,6]. Fetal and neonatal outcomes, including preterm delivery, birth weight, live birth, and neonatal ICU admission, were also not consistently available with sufficient completeness for reliable analysis. Future studies incorporating these outcomes would provide a more comprehensive understanding of the maternal-fetal implications of severe psoriasis during pregnancy [2,10].

Pregnancy-related physiological changes may influence leukocyte count, serum albumin, renal indices, hepatic parameters, and inflammatory markers, emphasizing the importance of pregnancy-specific interpretation of biochemical findings [5,13,18]. Although the hospitalization model demonstrated good discrimination within the present cohort, internal model performance does not establish transportability, prospective predictive performance, or clinical utility [3]. Independent external validation is therefore necessary before these clinical and biochemical factors can be incorporated into formal prediction or risk-stratification tools [19,20].

Larger prospective multicenter studies should assess whether psoriasis phenotype, together with inflammatory, nutritional, hepatic, and renal parameters, demonstrates consistent associations with escalation of care across different populations and healthcare systems [7,19]. Future studies should incorporate standardized admission criteria, detailed medication exposure, gestational-age-specific analyses, nutritional and socioeconomic factors, and maternal, fetal, and neonatal outcomes [2,20]. External validation in independent cohorts will also be required before combinations of these factors can be considered for formal clinical prediction or risk-stratification models [19,20].

## Conclusions

Generalized pustular or erythrodermic psoriasis, elevated CRP, and hypoalbuminemia were independently associated with hospitalization among pregnant women with severe psoriasis. In exploratory analyses among hospitalized participants, systemic infection, marked leukocytosis, hepatic dysfunction, AKI, electrolyte disturbance, and generalized pustular or erythrodermic phenotype were more frequent among women requiring ICU admission. Because only 17 ICU events occurred, these findings should be regarded as exploratory and hypothesis-generating rather than definitive.

Assessment of psoriasis phenotype, together with routinely available clinical and biochemical findings, may help identify patients who warrant closer observation and multidisciplinary evaluation. However, hospitalization and ICU admission are influenced by both clinical severity and healthcare-process factors, and the present findings do not constitute a validated prediction or risk-stratification model. Larger prospective multicenter studies with external validation and detailed treatment, maternal, fetal, and neonatal outcome data are required before these associations can be incorporated into formal clinical prediction tools.

## References

[1] Bar D, Pavlotsky F, Barzilai A, Yinon Y, Baum M (2026). Pregnancy outcomes in patients with biologic-exposed psoriasis: a large cohort study of real-world safety (IN PRESS). J Am Acad Dermatol.

[2] Mößner R, Cramer N, Gerdes S (2026). S1 guideline: therapy of generalized pustular psoriasis. J Dtsch Dermatol Ges.

[3] Puig L, Carrascosa JM, Rivera R, Vilarrasa E, de la Cueva P, López-Ferrer A (2026). Generalized pustular psoriasis: review and consensus of the psoriasis group of the Spanish Academy of Dermatology and Venereology. Actas Dermosifiliogr.

[4] Lee CC, Huang YH, Chi CC, Chung WH, Chen CB (2025). Generalized pustular psoriasis: immunological mechanisms, genetics, and emerging therapeutics. Trends Immunol.

[5] Choon SE, Foley PA, Asawanonda P (2024). Asia-Pacific consensus recommendations on the management of generalized pustular psoriasis. J Dermatol.

[6] Wang Y, Zhou C, Hou Y, Gao Y, Lu J, Yin Z (2024). Biologic treatment outcomes in generalized pustular psoriasis of pregnancy: a systematic review. Australas J Dermatol.

[7] Pogorelov D, Tschesche A, Balakirski G, Hofmann SC (2024). Generalized pustular psoriasis of pregnancy successfully treated with certolizumab pegol: a case report and literature review. Cureus.

[8] Rahmati S, Moameri H, Mohammadi NM (2023). Impact of maternal psoriasis on adverse maternal and neonatal outcomes: a systematic review and meta-analysis. BMC Pregnancy Childbirth.

[9] Puig L, Choon SE, Gottlieb AB (2023). Generalized pustular psoriasis: a global Delphi consensus on clinical course, diagnosis, treatment goals and disease management. J Eur Acad Dermatol Venereol.

[10] Sánchez-García V, Hernández-Quiles R, de-Miguel-Balsa E, Giménez-Richarte Á, Ramos-Rincón JM, Belinchón-Romero I (2023). Exposure to biologic therapy before and during pregnancy in patients with psoriasis: systematic review and meta-analysis. J Eur Acad Dermatol Venereol.

[11] Dajti E, Bruni A, Barbara G, Azzaroli F (2023). Diagnostic approach to elevated liver function tests during pregnancy: a pragmatic narrative review. J Pers Med.

[12] Seishima M, Fujii K, Mizutani Y (2022). Generalized pustular psoriasis in pregnancy: current and future treatments. Am J Clin Dermatol.

[13] Costanzo A, Bardazzi F, DE Simone C (2022). Pustular psoriasis with a focus on generalized pustular psoriasis: classification and diagnostic criteria. An Italian expert consensus. Ital J Dermatol Venerol.

[14] Krim D, Gomolin A, Czuzoj-Shulman N, Abenhaim HA (2022). Maternal and neonatal outcomes of births to women with psoriasis: a population-based cohort of 13 million births. J Matern Fetal Neonatal Med.

[15] Groenendijk W, Bogdanet D, Dervan L (2022). Reference intervals for clinical biochemistry and haematology tests during normal pregnancy. Ann Clin Biochem.

[16] Shalaby AS, Shemies RS (2022). Pregnancy-related acute kidney injury in the African continent: where do we stand? A systematic review. J Nephrol.

[17] Dockree S, Shine B, Pavord S, Impey L, Vatish M (2021). White blood cells in pregnancy: reference intervals for before and after delivery. EBioMedicine.

[18] Dockree S, Brook J, James T, Shine B, Impey L, Vatish M (2021). Pregnancy-specific reference intervals for C-reactive protein improve diagnostic accuracy for infection: a longitudinal study. Clin Chim Acta.

[19] Lambe M, Bergstrom AV, Johansson AL, Weibull CE (2020). Reproductive patterns and maternal and pregnancy outcomes in women with psoriasis - a population-based study. J Am Acad Dermatol.

[20] Odorici G, Di Lernia V, Bardazzi F (2019). Psoriasis and pregnancy outcomes in biological therapies: a real-life, multi-centre experience. J Eur Acad Dermatol Venereol.

